# Novel Multiscale Modeling Tool Applied to *Pseudomonas aeruginosa* Biofilm Formation

**DOI:** 10.1371/journal.pone.0078011

**Published:** 2013-10-17

**Authors:** Matthew B. Biggs, Jason A. Papin

**Affiliations:** Department of Biomedical Engineering, University of Virginia, Charlottesville, Virginia, United States of America; University of Osnabrueck, Germany

## Abstract

Multiscale modeling is used to represent biological systems with increasing frequency and success. Multiscale models are often hybrids of different modeling frameworks and programming languages. We present the MATLAB-NetLogo extension (MatNet) as a novel tool for multiscale modeling. We demonstrate the utility of the tool with a multiscale model of *Pseudomonas aeruginosa* biofilm formation that incorporates both an agent-based model (ABM) and constraint-based metabolic modeling. The hybrid model correctly recapitulates oxygen-limited biofilm metabolic activity and predicts increased growth rate via anaerobic respiration with the addition of nitrate to the growth media. In addition, a genome-wide survey of metabolic mutants and biofilm formation exemplifies the powerful analyses that are enabled by this computational modeling tool.

## Introduction

Multiscale modeling is a broad class of hybrid modeling techniques that attempt to represent physical systems that span multiple spatial or time scales. Spatial and time scales are particularly interdependent in biological applications and there is increasing utility for multiscale models that capture this interdependency [1]. A recent example is a model of vascular adaptation that combines an agent-based model (i.e. cellular level) with a continuum biomechanical model (i.e. tissue level) [2,3]. Using this model, Hayenga et al. identify causal factors in arterial adaptation to sustained increases in blood pressure. These predicted factors are active at different spatial scales and include cell growth and tissue remodeling. This remodeling in turn occurs as a function of the changes in production and removal of collagen and smooth muscle cells due to hemodynamically-induced stresses, emphasizing the highly multiscale nature of the biological system and the need for mathematical models that integrate data from disparate spatial and temporal scales [2].. Multiscale models show significant potential for representing the inherent complexity of biological systems, generating testable hypotheses to understand fundamental mechanisms.

The hybrid nature of many multiscale models creates a need for software tools in which to implement the models. Different software packages offer unique strengths (e.g. R provides vast statistics capabilities [4], NetLogo provides a rich environment for agent-based modeling [5], and MATLAB offers a wealth of engineering tools [6]). It is often advantageous to implement separate portions of a model in the most appropriate language and to combine the results dynamically. Dynamically combining model results between software platforms can be achieved with packages written for that purpose. Examples of current packages that perform this function are the NetLogo-R extension by Thiele and Grimm [7] and R.matlab by Bengtsson [8]. As multiscale models are built with increasingly diverse computational components, more tools will be needed that facilitate dynamic integration of disparate software tools.

Here, we present a novel software tool that fills a need in biomedical and biological multiscale modeling. The MATLAB-NetLogo extension (MatNet) provides new functions within NetLogo that allow data passing between NetLogo and MATLAB, and the calling of any valid, one-line MATLAB commands from within NetLogo. The need for this tool is demonstrated by publications that have used NetLogo and MATLAB (as the most appropriate software platforms) to implement biomedical multiscale models [2,3,9]. The new tool presented herein facilitates future dynamic integration of these software platforms.

To demonstrate the utility of this tool, we present a multiscale model of *Pseudomonas aeruginosa* biofilm growth. *P. aeruginosa* is a common opportunistic pathogen that forms biofilms on medical implants [10] and in the lungs of cystic fibrosis patients [11], and is a model organism for biofilm formation. In our model, we combine an existing ABM of biofilm development [12,13] with a genome-scale metabolic model of *P. aeruginosa* metabolism [14]. This biofilm model is multiscale in its incorporation of biofilm-level spatial information such as structural remodeling and nutrient diffusion, as well as cell-level details of metabolic functions such as nutrient uptake and growth yields. The ABM, originally developed in C++, was implemented in NetLogo to exploit the existing framework and flexibility it offers as an ABM platform [15]. Metabolic modeling was implemented in MATLAB as done previously [16]. The resulting model reproduces known biofilm structure from limited oxygen diffusion. The model further demonstrates the utility of MatNet by generating hypotheses for how gene-level perturbations influence biofilm structure.

## Methods

### Agent-Based Model of Biofilm Structure

Here, we briefly describe the structure and processes of the ABM and refer the reader to our publicly-available model as well as corresponding citations for further details. The rules for the two-dimensional ABM of biofilm growth were implemented in NetLogo essentially as described by Pizarro et al. [12,13]. The purpose of the ABM is to capture emergent biofilm structure that results from growth and dispersion of individual bacterial cells. The biofilm is represented as a two-dimensional cross-section divided into squares. Each square represents a region of liquid growth media. As such, each square contains variables that represent nutrient levels in that area, and nutrients are allowed to diffuse from higher to lower concentrations. Each agent in the simulation represents bacteria. Agents diffuse randomly unless adjacent to “biofilm”. “Biofilm” is defined in the simulation as agents directly adjacent to the bottom surface of the simulated space, or adjacent to a chain of agents that terminates at the bottom surface. Agents in the biofilm do not move except as a result of division. Bacterial agents undergo binary division once the nutrients consumed exceed a pre-defined threshold. Only one agent may occupy a square; therefore, once an agent divides into two, the new agent is placed in a randomly-selected adjacent square, and if that square is occupied, the next agent is displaced to a random adjacent square. This process, termed “shoving”, is continued until no square contains more than one agent. 

The key difference in our model from the Pizarro et al. formulation is a change from representative “food particles” to concentrations of all 105 extracellular metabolites used in the genome-scale metabolic network reconstruction of *P. aeruginosa* [14]. Each metabolite diffuses independently as a function of the molecular mass. Metabolites diffuse more slowly through regions of the ABM space defined as biofilm (60% of aqueous rate for gases, and 25% of aqueous rate for all other metabolites) [17]. 

The multiscale modeling of the biofilm is an iterative process involving analysis in MATLAB and NetLogo. First, constraints on exchange fluxes for the FBA problem in MATLAB are scaled to local nutrient concentrations. This simplifying assumption can be relaxed with more detailed flux constraints implemented as such data is available. However, these simplified constraints are sufficient to illustrate the value of the modeling tool presented here. After solving the FBA problem in MATLAB, local nutrient concentrations are calculated and returned, along with the growth rate, to the NetLogo environment. The nutrient concentrations are updated in NetLogo, agents with accumulated biomass divide in two and rearrange according to the shoving rule, nutrients diffuse, and the new nutrient concentrations are passed to MATLAB. These steps constitute one time step of the simulation, which simulates a 5 minute interval of biofilm growth. A single simulation of 200 time steps simulates biofilm growth over a period of ~17 hours. 

Our implementation of the biofilm model in NetLogo displays the same behavior as the Pizarro et al. model (Figure S1). Because the ABM was independently validated previously [12,13], it will not be further validated here except as pertains to the hybrid metabolic and agent-based models.

### Genome-Scale Metabolic Network Reconstruction

*P. aeruginosa* metabolism was modeled using the previously published genome-scale metabolic reconstruction [14]. The model was analyzed with functions from the COBRA Toolbox [18] implemented previously in MATLAB. The COBRA Toolbox utilized the Gurobi optimizer [19]. Metabolite concentrations in each occupied square of the ABM were used to constrain uptake rates in the model. Discrete solutions for each cell agent at each time point were found using flux balance analysis (FBA) [20]. Cell agent biomass and metabolite concentrations were updated using dynamic FBA [21]. 

### Metabolic Model Constraints

Initial conditions simulating glucose minimal media were generated by including negative, non-zero lower bounds for the extracellular metabolite exchange reactions: Iron (Fe and Fe_3+_), Oxygen (O_2_), D-Glucose (C_6_H_12_O_6_), Cadmium (Cd), Carbon Dioxide (CO_2_), Sulfate (H_2_O_4_S), Copper (Cu), Water (H_2_O), Manganese (Mn), Cobalt (Co), Ammonium (NH_4_+), Sodium (Na), Nitrogen (N_2_), Magnesium (Mg), Orthophosphate (H_3_O_4_P), and Zinc (Zn). For the anaerobic respiration simulation, an additional negative, non-zero lower bound was included for the Nitrate (HNO_3_) exchange reaction. The metabolic model and accompanying constraints were previously described [14] and were not further validated here except as pertains to the hybrid model.

### Software Availability

MatNet, example code, and the biofilm model are available from:

http://bme.virginia.edu/csbl/downloads.php

### Simulation Specifications

Simulations were performed on a 64-bit Sony Vaio laptop with 6 GB of RAM and a 2.8 GHz dual-core processor running Windows 7, NetLogo version 5.0.3 and MATLAB version 2012b. The duration of single simulations of biofilm growth ranged from 5 to 15 hours, depending on model settings. 

## Results and Discussion

### Novel Multiscale Modeling Tool

**Figure 1:**
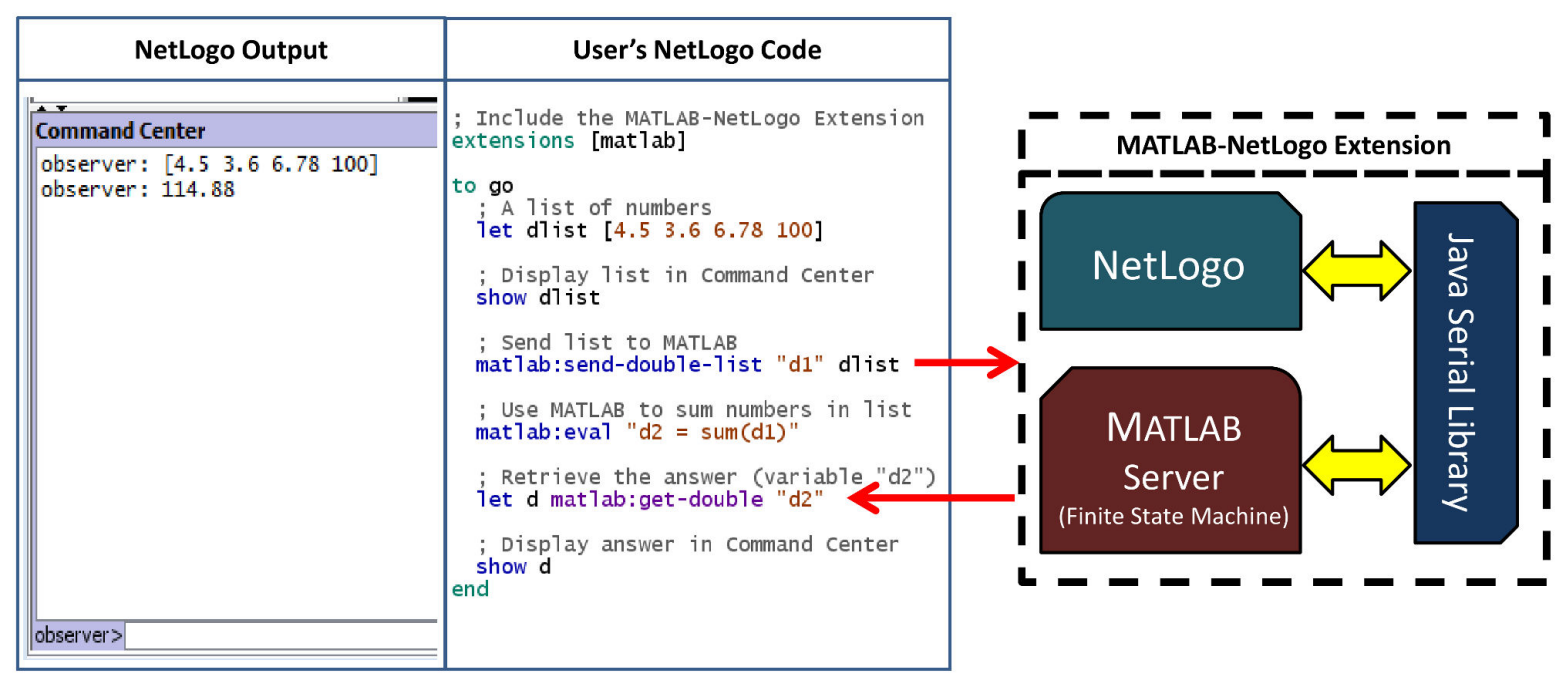
MATLAB-NetLogo Extension (MatNet) diagram and example code. MATLAB and NetLogo are both Java-based applications and are able to pass data via the Java Serial library. The user is insulated from the details of data passing, and can call MATLAB functions (native or user-defined) from within NetLogo using simple commands. In the example above, a list of numbers is created in NetLogo and passed to MATLAB where the numbers are summed. The answer is retrieved from MATLAB and displayed in NetLogo.

MatNet was written in Java, utilizing the NetLogo Extensions API (Figure 1). NetLogo and MATLAB pass data using the Java Serial library. MATLAB is opened as a background process and runs a server script that is an implementation of a finite state machine. The architecture was based on R.matlab [8] and the NetLogo-R extension [7]. This extension adds nine commands or “primitives” for sharing and evaluating data with MATLAB from within NetLogo (see “User Guide” in Material S1). The resulting extension provides a simple interface between the NetLogo and MATLAB platforms that allows users to exploit the strengths of both languages in their models (Figure 1). While the following multiscale analysis is a biomedical example, this tool could readily find application in other fields for which integrated MATLAB and NetLogo analyses are of value such as ecology [31], finance [32], or behavioral science [33].

Individual simulations were performed over 5 to 15 hours. We evaluated the computational time for each of the functions in a given simulation. A large fraction of the simulation run time is claimed by the metabolite diffusion simulations in NetLogo and the repeated FBA simulations in MATLAB. The slower run time of these steps is expected, given that both processes are called frequently during each time step, and both are computationally intensive. While an appreciable portion of the computational time was spent passing data between MATLAB and NetLogo, this computational time is attributable to the high frequency with which these functions were called. The passing of data between the two environments via MatNet did not add undue computational overhead. Among all the functions in the simulation, each MatNet function was listed among the fastest on a per-function-call basis.

### Oxygen-Limited Metabolic Activity in a *P. aeruginosa* Biofilm Model

**Figure 2:**
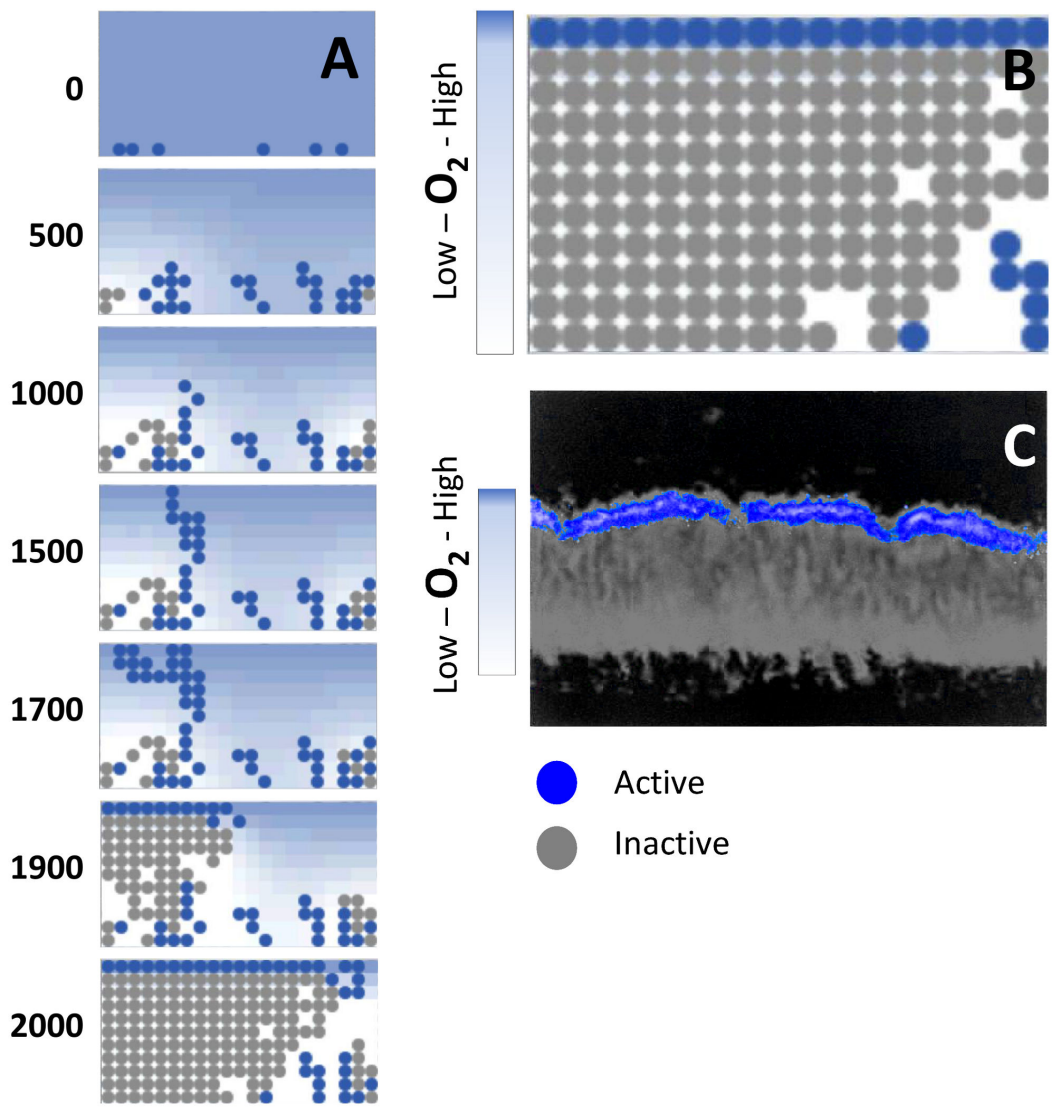
Oxygen-dependent metabolic activity in *P. aeruginosa* biofilms. (**A**) Progression of biofilm growth in a multiscale model with the associated time step (time steps represent 5 minute intervals). Each circle represents a cluster of *P. aeruginosa* cells. (**B**) Snapshot from multiscale biofilm model in glucose minimal media at time step 2000. (**C**) *in* *vitro* *P. aeruginosa* biofilm cross section grown in glucose MOPS media for four days (modified from Xu et al. [22]). The oxygen gradient through the biofilm limits metabolic activity. Only with high O_2_ (near the surface) can cells actively synthesize protein. The multiscale model recapitulates this pattern of oxygen-limited metabolic activity throughout the biofilm.

The ABM correctly recapitulates oxygen-limited metabolic activity in a biofilm. Biofilm formation was simulated under glucose minimal media conditions. Metabolic activity was defined as an increase in biomass (> 0.01 mass dry weight) associated with a particular agent in the two-dimensional space. Metabolites were allowed to diffuse in from the top to mimic fresh media being washed over the biofilm as done by Pizarro et al [12,13]. Oxygen at the top was held at a constant 0.21 mM [21]. All simulations showed reduced metabolic activity in the interior of the biofilm, and increased metabolic activity at the surface. An evaluation of the exchange reaction fluxes in the metabolic models indicated oxygen as the limiting metabolite (Figure 2B), consistent with findings from Xu et al. who report oxygen-limited growth in *P. aeruginosa* biofilms (Figure 2C) [22]. Furthermore, metabolic activity (as measured by protein synthesis) is restricted to a layer of cells at the biofilm surface (Figure 2C) as previously reported [22]. Therefore, this model of biofilm growth correctly recapitulated known characteristics of *P. aeruginosa* biofilm.

### Nitrate Promotes Anaerobic Respiration and Increased Biofilm Growth Rate

**Figure 3:**
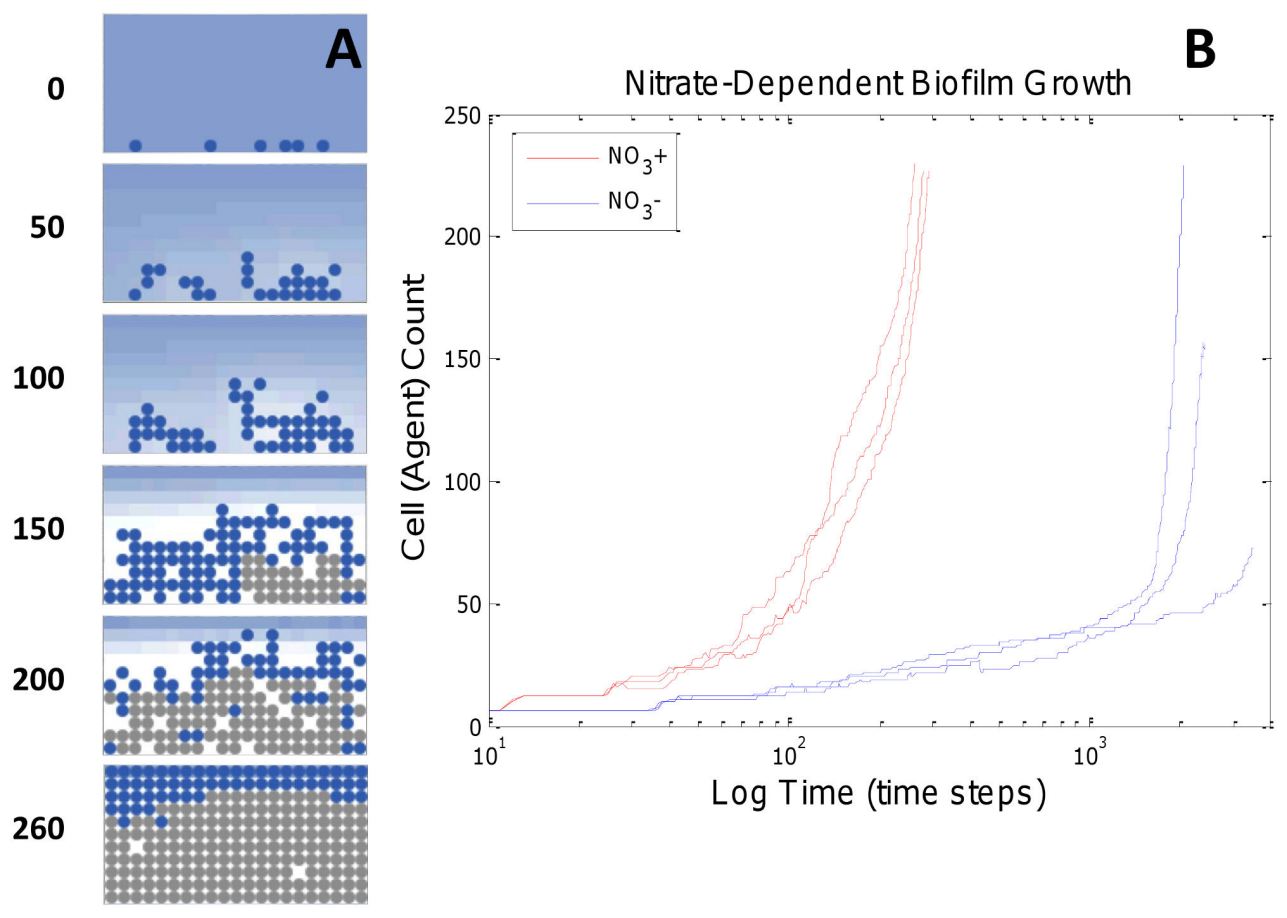
ABM simulations of nitrate-dependent growth rates. (**A**) Predicted biofilm formation in the presence of nitrate (NO_3_) shows higher proportion of active cells when compared to glucose minimal media control (Figure 2). (**B**) Predicted biofilm growth with and without nitrate (3 independent runs each). Addition of nitrate is predicted to increase biofilm growth rate by enabling anaerobic growth deeper in the biofilm. Note that for simulations in glucose minimal media (blue lines), slower growth increases the impact of random cell spacing and resultant heterogeneous nutrient usage such that the model resulted in differing final cell counts for the same 15 hour simulation times.

Our multiscale model recapitulated increased biofilm growth rate in nitrate-supplemented media. Addition of nitrate (NO_3_) to the *in silico* growth media increased biofilm growth rate by approximately 10-fold, as determined by the change in cell agent counts over the first 263 time steps (Figure 3B). Nitrate relieves the oxygen limitation in *P. aeruginosa* by allowing anaerobic growth via denitrification [22,23]. Denitrification, or anaerobic respiration, is the process whereby nitrate (NO_3_) is reduced to dinitrogen (N_2_), and nitrate replaces gaseous oxygen as the terminal electron acceptor. Anaerobic respiration prolongs active growth deeper in the biofilm after oxygen is removed from the microenvironment. The model prediction of increased growth rate was subsequently validated via literature search; Borriello et al. report increased biofilm growth with the addition of nitrate [24]. Although a direct comparison is not possible due to different growth conditions than those simulated in the model, the results reported by Borriello et al. serve as a qualitative validation for the model predictions. This validated model prediction demonstrates that hybrid ABM-metabolic models can display predictive emergent behavior that is physiologically relevant. 

### *in silico* Gene-Deletion Screen

**Figure 4:**
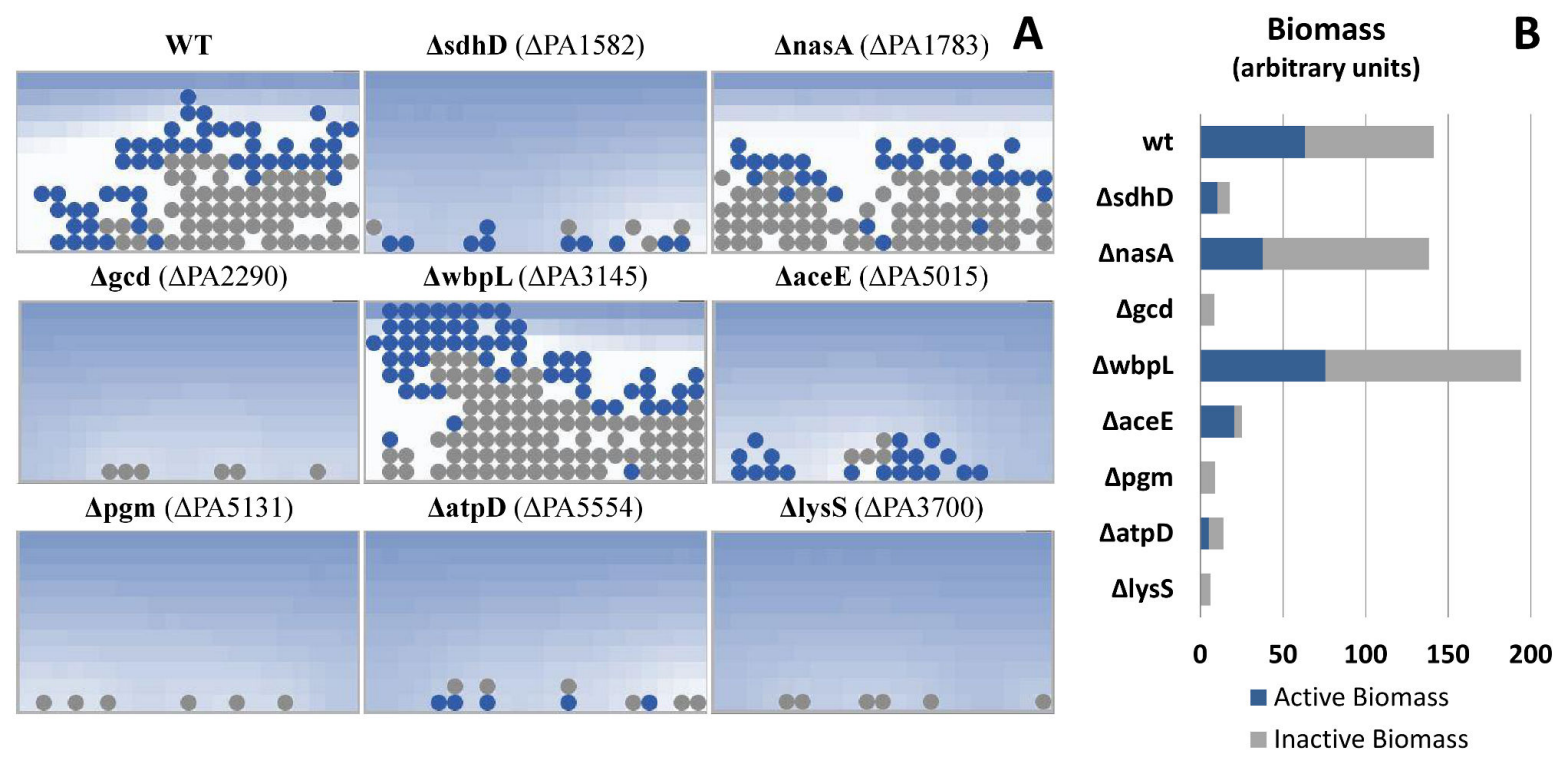
Single-gene deletion screen. Models of several single-deletion mutants were evaluated for biofilm formation after 200 time steps in nitrate-supplemented glucose minimal media. The wild-type (WT) model serves as a positive control. Δ*lysS* is known to be lethal, and provides a negative control. As such, the six initial cells seeded in the model never produced any additional biomass. (**A**) Snapshots of each multiscale simulation at time step 200. (**B**) Proportions of active and inactive biomass for each ABM at time step 200. Δ*sdhD*, Δ*aceE* and Δ*atpD* grew more slowly than wild-type. Δ*gcd* and Δ*pgm* appeared to have significant growth defects (final biomass only slightly more than that initially seeded). This screen is an example of a powerful analysis that is enabled by the multiscale simulations integrating spatial modeling with NetLogo and the metabolic network analysis performed in MATLAB.

An *in silico* gene-deletion screen predicts the influence of individual genes on biofilm growth. Genes were deleted from the metabolic model by constraining reaction flux to zero. All possible single-gene deletions were evaluated in MATLAB using FBA. From the results of this analysis, a subset of metabolic models was selected to represent a range of growth phenotypes (lethal, sub-optimal and wild-type). A multiscale model was generated for each mutant background selected and was evaluated for 200 time steps on nitrate-supplemented glucose minimal media (Figure 4). Qualitative behavior was clearly evident by time step 200, which was chosen consequently as a stopping point. Note that with MatNet a genome-wide gene deletion screen and the resulting phenotypic differences of a multicellular system can quickly and easily be explored, thus providing useful hypotheses to guide experimental design.

We present the hybrid model results for nine models: wild-type, Δ*sdhD*, Δ*nasA*, Δ*gcd*, Δ*wbpL*, Δ*aceE*, Δ*pgm*, Δ*atpD*, and Δ*lysS*. The wild-type model served as a positive control, while Δ*lysS* served as a negative control (*lysS* encodes a tRNA synthetase and is an essential gene on nitrate-supplemented glucose minimal media). Reduced growth was predicted for Δ*sdhD*, Δ*aceE* and Δ*atpD*. *sdhD* plays a role in aerobic respiration [25] and its deletion restricts growth by limiting cells to anaerobic respiration. *atpD* encodes a subunit of ATP synthase. *aceE* encodes a pyruvate dehydrogenase and its deletion uncouples the citric acid cycle from glycolysis. Severely restricted growth (only slightly more biomass was found at time step 200 than what was initially seeded into the system) was predicted for Δ*gcd* and Δ*pgm*. *gcd* encodes a glucose dehydrogenase and de Werra et al. report that on glucose minimal media, mutant strains without *gcd* initially grow very slowly [26]. *pgm* encodes a phosphoglycerate mutase. The Δ*nasA* model is of interest because *nasA* encodes a nitrate transporter, and yet the model predicts near-wild-type growth on nitrate-supplemented media. Further investigation showed that the metabolic reconstruction contains two independent nitrate transport pathways. In the Δ*nasA* model, nitrate is taken into the cell via a separate nitrate ABC transporter encoded by PA2294, PA2295, PA2296, or PA2327, PA2328, PA2329. The results of the Δ*nasA* model are of further interest because they highlight the utility of this multiscale modeling approach to explore the interplay of gene function and biofilm microenvironment heterogeneity. While some model predictions were validated through literature search, the unsupported predictions stand as hypotheses awaiting experimental validation. The purpose of this screen is simply to demonstrate the power of our hybrid model to survey genome-wide, gene-level perturbations on biofilm-level phenotype.

## Conclusion

This model framework correctly recapitulated known biofilm characteristics and yielded useful predictions that may guide future experimental design. Future development of the models presented here could include an accounting of extracellular polymeric substances in the ABM [27–30], the addition of rules linking specific genes to biofilm growth, and the inclusion of gene regulation in the metabolic model. Another potential biological process highly amenable to hybrid modeling using MatNet is quorum sensing, in which spatial information of the cells contributes to the signaling and gene regulation of the bacteria. Models of quorum sensing could also be integrated with the biofilm model, facilitating an interrogation of the transition from a planktonic to biofilm state. The current work demonstrates that even simplified multiscale models can capture important biological behaviors that would be difficult or impossible to predict otherwise, and that our tool enables powerful cross-platform modeling that could be of value in multiple biomedical and other applications.

## Supporting Information

Figure S1 **Oscillating biofilm thickness.** Our implementation of the agent-based model as described by Pizarro et al demonstrates the same oscillatory behavior that they report. This is due to the degradation of the lower levels of biofilm over time, which eventually causes entire segments of biofilm to slough off, leading to cyclic variation in biofilm thickness.
(TIF)

Material S1 **The User Guide provides detailed instructions for installation and use of MatNet.**
(DOCX)
