# Supplementary figures and images for: Novel Multiscale Modeling Tool Applied to Pseudomonas aeruginosa Biofilm Formation
Source: PLoS One. 2013 Oct 17;8(10):e78011. doi: 10.1371/journal.pone.0078011 (PMC3798466; doi:10.1371/journal.pone.0078011)

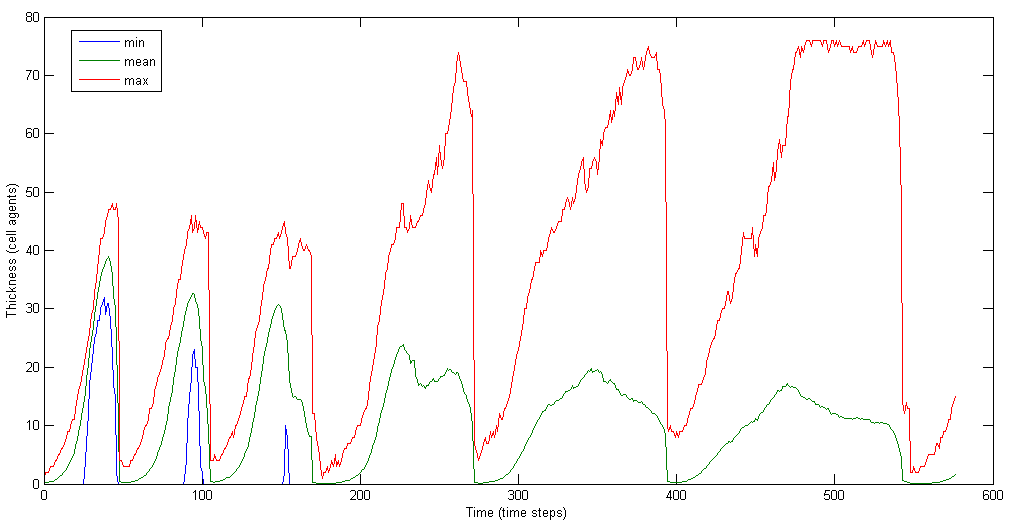

Supplement: Figure S1 — Oscillating biofilm thickness. Our implementation of the agent-based model as described by Pizarro et al demonstrates the same oscillatory behavior that they report. This is due to the degradation of the lower levels of biofilm over time, which eventually causes entire segments of biofilm to slough off, leading to cyclic variation in biofilm thickness. (TIF) [file pone.0078011.s001.tif]
