# Supplementary material for: Novel Multiscale Modeling Tool Applied to Pseudomonas aeruginosa Biofilm Formation
Source: PLoS One. 2013 Oct 17;8(10):e78011. doi: 10.1371/journal.pone.0078011 (PMC3798466; doi:10.1371/journal.pone.0078011)
Supplement: Material S1 — The User Guide provides detailed instructions for installation and use of MatNet. (DOCX) [file pone.0078011.s002.docx]

**Supplementary Material**

**MatNet User Guide:**

**Getting set up:**

1. You will need to have NetLogo and MATLAB installed before use of this extension.
2. Download the NetLogo MATLAB Extension as a ZIP file. Unzip it.
3. Place the folder ("matlab") containing "matlab.jar" in your "NetLogo_v#/extensions/" directory.
4. Place the file "matlabServer.m" in your MATLAB path.
5. To include the matlab extension in a netlogo script, place this at the head of your script:

extensions[matlab]

When you include the MATLAB extension, NetLogo will pause while MATLAB starts up. A MATLAB window will be opened (unless you're running Linux), and you will be able to see the output from the server. This window can be minimized and ignored. However, you will need to close these windows when you're done.

Because NetLogo only has two basic data types (Strings/Lists of strings and Doubles/Lists of Doubles) that are MATLAB-compatible, the functions provided by this extension only deal with passing those data types back and forth between NetLogo and MATLAB. All variables passed to MATLAB, or results of "eval" statements, persist in the MATLAB environment between commands, and can be re-accessed.

**The provided functions follow:**

*eval*

**Description:** eval allows the user to run any valid MATLAB command from NetLogo, including *.m files and custom functions/packages in the MATLAB path.

**Input:** Valid MATLAB command as a String.

**Output:** None.

**Example Usage:**

matlab:eval "a = 1 + 1"

matlab:eval "c = a / 32.2"

matlab:eval "b = {'efg' ; 'hi12'}"

matlab:eval "someFunction()"

**Note:** According to the MATLAB documentation, the eval function cannot create variables. These simple examples (e.g. "a = 1 + 1") _will_ create the variables such as "a" that can then be retrieved from within NetLogo. The difficulty is in getting values from your functions or scripts. The best workaround so far is to use the MATLAB *assignin* command to create variables in the workspace, rather than pass them back. You may find that NetLogo cannot run a function that was written after the extension was started (will print the error "empty command stream" in the MATLAB window). If this happens, close NetLogo, write and save your function, then re-open NetLogo.

Try this example sequence:

In a MATLAB function in your MATLAB path:

function [] = test_function(testInt)

testMat = magic(testInt);

testEigs = eig(testMat);

assignin('caller','retMat',testMat);

assignin('caller','retEigs',testEigs);

end

In NetLogo Script:

extensions[ matlab ]

globals [myEigs]

In NetLogo Command Center:

matlab:send-double "sendInt" 3

matlab:eval "test_function(sendInt)"

set myEigs matlab:get-double-list "retEigs"

show myEigs

**2nd Note:** Matrix passing is not currently supported by this extension, although a workaround has been suggested: it would be possible to use the "get-double-list" or "send-double-list" command within a loop structure to grab/send each column or row in your target matrix.

**3rd Note:** Currently, if the function sent to MATLAB (using the "eval" command) is slow, NetLogo will not wait for the results. If you need NetLogo to wait until MATLAB is done, one possible workaround is to do something like the code below using a wait command within a loop. In this example, I set a limit on the number of iterations I'm willing to wait:

matlab:eval "a=1;"

matlab:eval "slowFunction();a=a+1;"

; Don't continue until slowFunction() is done

set matlabReady? false

let mlcount 0

while [ matlabReady? = false ]

[

set matlabReturnVal (matlab:get-double "a")

if matlabReturnVal = 2

[

set matlabReady? true

]

if mlcount > 50

[

set matlabReady? true

]

set mlcount (mlcount + 1)

wait 0.1

]

*get-double*

**Description:** Returns a "double" stored in the MATLAB environment (if it exists).

**Input:** The name for that variable as it appears in MATLAB.

**Output:** Double.

**Example Usage:**

set myNumber matlab:get-double "varName"

*get-double-list*

**Description:** Returns a list of "doubles" stored in the MATLAB environment (if it exists).

**Input:** The name for that variable as it appears in MATLAB.

**Output:** List of doubles.

**Example Usage:**

set myList matlab:get-double-list "varName"

*get-string*

**Description:** Returns a "string" stored in the MATLAB environment (if it exists).

**Input:** The name for that variable as it appears in MATLAB.

**Output:** String.

**Example Usage:**

set myString matlab:get-string "varName"

*get-string-list*

**Description:** Returns a list of "strings" stored in the MATLAB environment (if it exists).

**Input:** The name for that variable as it appears in MATLAB.

**Output:** List of strings.

**Example Usage:**

set myString matlab:get-string-list "varName"

*send-double*

**Description:** Passes a variable of type "Double" to MATLAB.

**Input:** Variable of type double (just a NetLogo number), and a name for that variable to be stored under in the MATLAB environment.

**Output:** None.

**Example Usage:**

matlab:send-double "varName" 123.4

*send-double-list*

**Description:** Passes a list of variables of type "Double" to MATLAB.

**Input:** List of variables of type double (just NetLogo numbers in a list), and a name for that variable to be stored under in the MATLAB environment.

**Output:** None.

**Example Usage:**

matlab:send-double-list "varName" (list 13.4 3.14798 1 2)

*send-string*

**Description:** Passes a variable of type "String" to MATLAB.

**Input:** String variable, and a name for that variable to be stored under in the MATLAB environment.

**Output:** None.

**Example Usage:**

matlab:send-string "varName" "This is my 1st string."

*send-string-list*

**Description:** Passes a list of "Strings" to MATLAB.

**Input:** List of "strings", and a name for that variable to be stored under in the MATLAB environment.

**Output:** None.

**Example Usage:**

matlab:send-string-list "varName" (list "a" "Billy" "This is my 1st string.")

**Versions:**

Version 1.0: February 2013. Tested with NetLogo 5.0.3, 5.0.4 and MATLAB R2012a, R2013a on Windows 7 and Ubuntu 12.04.
